# Anti-inflammatory effects of enzymatic hydrolysates of velvet antler in RAW 264.7 cells in vitro and zebrafish model

**DOI:** 10.17179/excli2015-481

**Published:** 2015-10-20

**Authors:** Seung-Hong Lee, Hye-Won Yang, Yuling Ding, Yanmei Wang, You-Jin Jeon, Sang-Ho Moon, Byong-Tae Jeon, Si-Heung Sung

**Affiliations:** 1 Division of Food Bioscience and Korea Nokyong Research Center, Konkuk University, Chungju, 380-701, Korea; 2 Department of Marine Life Science, Jeju National University, Jeju 690-756, Korea; 3 Jilin Sino-Rok Institue of Animal Science, Changchun 130-600, China

**Keywords:** enzymatic hydrolysis, velvet antler, bioactive components, anti-inflammatory effect

## Abstract

Enzymatic hydrolysis has been successfully used for the extraction of numerous biologically active components from a wide variety of natural sources. In the present study, velvet antler was subjected to the extraction process using Alcalase protease. We analyzed bioactive components, such as uronic acid, sulfated-glycosaminoglycans (sulfated-GAGs), and sialic acid, present in the velvet antler Alcalase hydrolysate (VAAH) and assessed their anti-inflammatory effects in zebrafish as well as *in vitro* using cell lines. VAAH mainly contained uronic acid (78.22 mg/g) and sulfated-GAGs (50.47 mg/g), while the amount of sialic acid was negligible (5.55 mg/g). VAAH inhibited the production of nitric oxide (NO) by lipopolysaccharide (LPS)-induced cells in a dose-dependent manner and the inhibitory effect of VAAH on NO production was higher than that of hot water extracts. VAAH treatment also reduced the expression of inflammatory mediators such as nitric oxide synthase (iNOS) and cyclooxygenase-2 (COX-2). Furthermore, we evaluated anti-inflammatory effects of VAAH using LPS-stimulated zebrafish. Treatment with LPS significantly increased cell death, NO, and reactive oxygen species (ROS) levels in zebrafish. Notably, VAAH significantly inhibited the extent of LPS-stimulated cell death and generation of NO and ROS in zebrafish. These results suggest that VAAH alleviated inflammation and cell death by inhibiting the generation of ROS induced by LPS treatment. Thus, VAAH could be used as a potential natural remedy with a strong anti-inflammatory effect. Taken together, we believe that based on our present results, enzymatic hydrolysis of velvet antler may be an effective process to make antler products acceptable as elements of health foods and nutraceutical components with increased biological activity.

## Introduction

Inflammation is a complex physiological response of the body to cell damage and tissue vascularization. Inflammatory responses are controlled by cytokines, products of plasma enzyme systems, and lipid mediators (Ross and Auger, 2002[25]). However, excessive and uncontrolled inflammation is deleterious to all tissues, as it may cause many acute and chronic human diseases (Medzhitov and Janeway, 1997[21]; Serhan and Savill, 2005[27]). Therefore, inhibition of excessive inflammation is important for reducing the risk of inflammation-derived diseases. Recently, there has been an increasing awareness of benefits of health foods from natural products and the interest in the discovery of phytochemical compounds has risen exponentially. Many studies identified natural anti-inflammatory agents derived from herbs, spices, and plants (Kazlowska et al., 2010[12]; Mueller et al., 2010[22]; Heo et al., 2012[8]). However, reports that document activity of health foods containing unique animal ingredients are still relatively rare. This is surprising because various bioactive components are present in animal tissues and they can be used in the prevention and treatment of many human diseases (Chen et al., 2010[2]; Ge et al., 2012[5]; Kim et al., 2014[13]).

Velvet antler is a typical traditional animal medicine that has been used for over 2000 years. It is considered to have various pharmacological effects including stimulation of the immune system, increase in the physical strength, and enhancement of sexual function (Kawtikwar et al., 2010[11]; Gilbey and Perezgonzalez, 2012[6]). Nowadays, velvet antler is recognized as a traditional medicine in pharmacopeias of Korea, China, and Japan. In addition, velvet antler is also used as a health food supplement and a nutraceutical in many countries, including East Asia, New Zealand, Canada, and USA, to prevent diseases presumably by actions of its diverse biological components (Sui et al., 2014[30]).

 Traditionally, main formulations of velvet antler are decoction and medicinal liquor, which have been used for long time. At the same time, many reports have shown that preparations of velvet antler obtained by extractions with hot water or ethanol exhibited different activities (Kim et al., 2003[15]; Je et al., 2010[10]). However, this approach is somewhat controversial, largely because of strict restrictions on the use of organic solvents in the food industry and a restricted recovery of water-soluble components in water extractions (Lee et al., 2012[19]). These extraction methods also could denature proteins and impair their activities (Sui et al., 2014[30]). Therefore, to make antler products acceptable as health foods and nutraceuticals, an effective and beneficial method of extraction of biological components from velvet antler has to be thoroughly investigated. Biochemists use various techniques to extract bioactive components from natural products. One of such techniques, enzymatic biomass hydrolysis, possesses certain benefits over other conventional techniques. Enzymes can transform water-insoluble materials into water-soluble ingredients. In addition, this method does not use any toxic chemicals. Interestingly, this technique allows extracting bioactive components with a high yield and often the extracted components show enhanced biological activity in comparison with products extracted with water and organic solvents (Lee et al., 2012[17]; Puri et al., 2012[24]; Ko et al., 2013[16]). 

 However to the best of our knowledge, there have been no reports about bioactive components that can be extracted from velvet antler by enzymatic hydrolysis. Thus, the objective of the present work was to analyze active components of the velvet antler hydrolysate and to study their anti-inflammatory effects *in vitro* and *in vivo*.

## Material and Methods

### Materials

Velvet antler was kindly provided by the Daesungsan Deer Farm (Daegwallyeong, Korea). It was obtained from farmed elk 75 days after casting. Fresh velvet antler was immediately sliced using a bone slicer and frozen at -20 °C. Frozen velvet antler slices were dried with a freeze dryer (Ilshin Lab Co., Ltd, Korea) and then ground into a fine powder by a grinder (HMF-3100S, Hanil Co., Korea). All chemicals and reagents used were of analytical grade and obtained from commercial sources.

### Preparation of hot water extract of velvet antler

For hot water extraction using dried velvet antler, 1 g of the ground velvet antler powder was mixed with 100 ml of water and placed into a shaking incubator for 24 h at 70 °C. The mixtures were centrifuged at 3000 × *g* for 20 min at 4 °C and filtered with Whatman filter paper to remove the residues.

### Preparation of enzymatic hydrolysate of velvet antler

Velvet antler enzymatic hydrolysis was performed according to the previously reported method (Ko et al., 2013[16]). Six gram of the ground dried velvet antler powder was homogenized with 600 ml of distilled water (pH 8.0) and 60 μl of Alcalase (Novo Nordisk, Bagsvaerd, Denmark) was added. Enzymatic hydrolysis was conducted for 24 h at 50 °C. As soon as the enzymatic reaction had been completed, the hydrolysate was boiled for 10 min at 100 °C to inactivate the enzyme. The hydrolysate was clarified by centrifugation at 3000 × *g* for 20 min to remove any unhydrolyzed residue. The supernatant of the velvet antler Alcalase hydrolysate (VAAH) was filtered, adjusted to pH 7.0, and stored for subsequent use in experiments.

### Analysis of bioactive components 

Sulfated-glycosaminoglycans (sulfated-GAGs) content was determined by the dimethylmethylene blue (DMB) dye assay method (Farndale et al., 1986[3]). Briefly, the color reagent was prepared by dissolving 0.008 g of DMB in a solution containing 1.185 g NaCl, 1.520 g glycine, 0.47 ml of 12 M HCl dissolved in 500 ml of distilled water. Each sample was mixed with 1 ml of this color reagent, and the absorbance was read immediately at 525 nm.

Uronic acid content was determined by the carbazole reaction (Cesaretti et al., 2003[1]). A 50-μl standard (carbazole) or samples were placed in a 96-well plate and supplemented with 200 μl of 25 mM sodium tetraborate solution in sulfuric acid. The plate was heated for 10 min at 100 °C in an oven. After cooling the plate for 15 min at room temperature, we carefully added 50 μl of 0.125 % carbazole dissolved in absolute ethanol. After a repeated round of heating at 100 °C for 10 min in the oven and cooling at room temperature for 15 min, the plate was read using a micro titer plate reader at a wavelength of 550 nm.

The sialic acid content was determined by the method of Warren (1959[32]) with a slight modification. Briefly, samples were hydrolyzed in 0.1 M H_2_SO_4_ in a final volume of 1 ml for 1 h at 80 °C. Both the standard and samples were incubated with 1 ml periodate solution at 37 °C for 30 min. After addition of 0.25 ml of 0.32 M sodium thiosulfate solution, the tubes were shaken until the characteristic yellow-brown color disappeared. The reaction was completed by the addition of 1.25 ml of 0.1 M thiobarbituric acid (TBA) solution and using a repeated cycle of heating the tubes to 100 °C for 15 min and cooling them to room temperature. The product was extracted with acidic butanol and optical density was determined at 549 nm.

### Cell culture

The murine macrophage cell line RAW 264.7 was purchased from the Korean Cell Line Bank (KCLB; Seoul, Korea). RAW264.7 cells were cultured in the Dulbecco's modified Eagle's medium (DMEM; GIBCO Inc., NY, USA) supplemented with 100 U/ml penicillin, 100 μg/ml streptomycin and 10 % FBS. The cells were then incubated in an atmosphere of 5 % CO_2_ at 37 °C and subcultured every 3 days.

### Determination of cell viability

Cell viability was measured using the conventional 3-(4,5-dimethyl-2-thiazolyl)-2,5-diphenyltetrazolium bromide (MTT) assay. RAW 264.7 cells were seeded in 96-well plates at a concentration of 1.5 × 10^5^ cells/ml. After 16 h, the cells were treated with lipopolysaccharide (LPS; 1 μg/ml) and the sample, followed by an additional incubation for 24 h at 37 °C. MTT stock solution (2 mg/ml) was added to wells for a total reaction volume of 200 μl. After a 4-h incubation period, the plates were centrifuged for 5 min at 800 × *g* and the supernatants were aspirated. Formazan crystals in each well were dissolved in 150 μl of dimethyl sulfoxide (DMSO) and the absorbance was measured by a microplate reader at a wavelength of 540 nm. 

### Nitric oxide (NO) production measurement

Cells were plated at a density of 1.5 × 10^5^ cells/well in 24-well plates and then incubated with or without LPS (1 μg/ml) in the absence or presence of various concentrations of the sample for 24 h. In brief, 100 μl of cell culture medium was mixed with 100 μl of Griess reagent (1 % sulfanilamide and 0.1 % naphthylethylenediamine dihydrochloride in 2.5 % phosphoric acid). The mixture was then incubated at room temperature for 10 min and the absorbance at 540 nm was measured by a microplate reader. Fresh culture media were used as blanks for all experiments. Nitrite levels in samples were determined using a standard sodium nitrite curve.

### Western blot analysis

RAW 264.7 cells (1.0 × 10^6^ cells/mL) were treated with LPS (1 μg/ml) and the sample for 24 h and cellular proteins were extracted from the cells. Protein concentrations were determined using a Bio-Rad protein assay kit (Bio-Rad, CA, USA) with bovine serum albumin (BSA) as a standard. Cell lysates (30-50 μg) were separated by SDS-PAGE (8-12 %) and the separated proteins were transferred to PVDF membranes (Bio-Rad) for 2 h. The membranes were pre-incubated with the blocking solution (5 % skim milk in Tris-buffered saline containing Tween-20) at room temperature for 2 h and then incubated with an anti-mouse iNOS antibody (1:1000; Calbiochem, La Jolla, CA, USA) and an anti-mouse COX-2 antibody (1:1000; BD Biosciences Pharmingen, San Jose, CA, USA) for 2 h at room temperature. After washing, the blots were incubated with horseradish peroxidase conjugated goat anti-mouse IgG secondary antibody (1:5000; Amersham Pharmacia Biotech, Little Chalfont, UK) for 30 min. The bands were visualized by chemiluminescence on X-ray films using ECL detection reagent (Amersham Biosciences, Piscataway, NJ, USA).

### Origin and maintenance of parental zebrafish

Adult zebrafish were obtained from a commercial dealer (Seoul aquarium, Seoul, Korea) and 10 fishes were kept in a 3-l acrylic tank with the following conditions; at 28.5 °C with a 14:10 h light:dark cycle. Zebrafish were fed three times a day, 6 days/week, with Tetramin flake food supplemented with live brine shrimps (*Artemia salina*; SE WHA PET food Co., Seoul, Korea). In the morning (on set of light), embryos were obtained from natural spawning. The collection of embryos was completed within 30 min.

### Evaluation of cell death and generation of intracellular reactive oxygen species (ROS) and nitric oxide (NO) in zebrafish embryo

Synchronized zebrafish embryos were collected and arrayed by a pipette in 12-well plates. Each well contained 2 ml of the embryo medium with 15 embryos during 7-9 h post-fertilization (hpf). Then the embryos were incubated with or without test samples for 1 h. To induce inflammation, the embryos were exposed to 10 µg/ml LPS dissolved in the embryo medium for 24 hpf at 28.5 °C. Thereafter, zebrafish embryos were transferred into fresh embryo medium, where they developed for up to 2 days post-fertilization (dpf). Cell death, intracellular ROS and NO generation in zebrafish embryos were estimated according to methods described by Lee et al. (2013[18]) and Kim et al. (2014[14]). Acridine orange is a nucleic acid selective fluorescent cationic dye useful for staining necrotic or very late apoptotic cells. Cell death was detected in live embryos using acridine orange staining. The zebrafish embryos were transferred into 24-well plates and treated with acridine orange solution (7 µg/ml), and the plates were incubated for 30 min in the dark at 28.5 °C. After incubation, the embryos were rinsed with fresh embryo media and anesthetized before observation and observed under a fluorescence microscope, which was equipped with a CoolSNAP-Pro color digital camera (Olympus, Tokyo, Japan). The images of stained embryos were analyzed for cell death and fluorescence intensity of individual embryos was quantified using ImageJ 1.46r software (Wayne Rasband, National Institutes of Health, Bethesda, MD, USA). Cell death were calculated by comparing the fluorescence intensity of treatment embryos to the controls. 

ROS generation in zebrafish embryos was detected using an oxidation-sensitive fluorescent probe dye, 2',7'-dichlorodihydrofluorescein diacetate (DCF-DA). The zebrafish embryos were transferred into 24-well plates and treated with DCF-DA solution (20 µg/ml), and the plates were incubated for 1 h in the dark at 28.5 °C. After incubation, the embryos were rinsed with fresh embryo media and anesthetized before observation and observed under a fluorescence microscope, which was equipped with a CoolSNAP-Pro color digital camera (Olympus, Tokyo, Japan). The images of stained embryos were analyzed for ROS generation and fluorescence intensity of individual embryos was quantified using ImageJ 1.46r software (Wayne Rasband, National Institutes of Health, Bethesda, MD, USA). Generation of ROS were calculated by comparing the fluorescence intensity of treatment embryos to the controls.

NO generation in zebrafish embryos was detected using a fluorescent probe dye, diamino-fluorophore 4-amino-5-methylamino-2',7'-difluorofluorescein diacetate (DAF-FM DA). The zebrafish embryos were transferred into 24-well plates and treated with DAF-FM DA solution (5 µM), and incubated for 1 h in the dark at 28.5 °C. After incubation, the embryos were rinsed with fresh embryo media and anesthetized before observation and observed under a fluorescence microscope, which was equipped with a CoolSNAP-Pro color digital camera (Olympus, Tokyo, Japan). The images of stained embryos were analyzed for NO generation and fluorescence intensity of individual embryos was quantified using ImageJ 1.46r software (Wayne Rasband, National Institutes of Health, Bethesda, MD, USA). Generation of NO were calculated by comparing the fluorescence intensity of treatment embryos to the controls.

### Statistical analysis 

The data are presented as the mean ± standard error. Statistical comparisons were performed using the SPSS package for Windows (Version 14). Differences between groups were considered significant if *P *< 0.05. 

## Results and Discussion

### Analysis of VAAH bioactive components

Traditional extraction of bioactive components derived from velvet antler generally utilized hot water. However, there is some controversy surrounding this approach, largely because of the extremely limited recovery of water-soluble components in water extractions. Recently, enzymatic extraction has been successfully applied for the extraction of numerous biologically active compounds from a great variety of natural products. This technique results in high yields of bioactive compounds, which show enhanced biological activity compared with ingredients extracted by water or organic solvents (Lee et al., 2012[17]; Puri et al., 2012[24]; Ko et al., 2013[16]). Therefore, in the present study, velvet antler was enzymatically hydrolyzed by using commercial proteases such as Alcalase (VAAH) to make antler products acceptable as health foods and nutraceuticals. 

The extraction yield and the list of bioactive components of VAAH are shown in Table 1(Tab. 1). The total extraction yield from VAAH was 32 %, which was higher than the extraction yield achieved with the hot water extract (25 %). With regards to bioactive components, uronic acid content in VAAH comprised 78.22 mg/g and was, therefore, higher than concentrations of other extractable components. VAAH also contained a significant quantity of sulfated-GAGs (50.47 mg/g) with minor amounts of the sialic acid (5.55 mg/g). These results suggest that the major bioactive component of VAAH is uronic acid, an acidic polysaccharide. It has been reported that uronic acid content was relatively higher than quantities of other bioactive components in velvet antler (Sunwoo et al., 1995[31]; Je et al., 2010[10]). When compared to previously published hot water extracts data (Je et al., 2010[10]), the sialic acid content of VAAH obtained in this study was similar to that observed in hot water extracts. However, sulfated-GAGs and uronic acid content were higher than those from hot water extracts. Taken together, these data indicate that enzymatic extraction techniques for the extraction of bioactive components of velvet antler may be more advantageous than hot water extraction.

### Effects of VAAH on LPS-induced NO production and cytotoxicity 

Macrophages produce NO in response to bacterial LPS and NO production can be controlled by a selective pharmacological inhibition of distinct nitric oxide synthase isoforms (Southan and Szabo, 1996[28]; Sarkar et al., 2008[26]). NO plays a pivotal role in many body functions, however, its overproduction, especially in macrophages, can lead to cytotoxicity, inflammation, and inflammation-derived diseases (Liu and Hotchkiss, 1995[20]). Therefore, inhibition of NO can provide an effective strategy for preventing inflammatory diseases.

**Figure 1:**
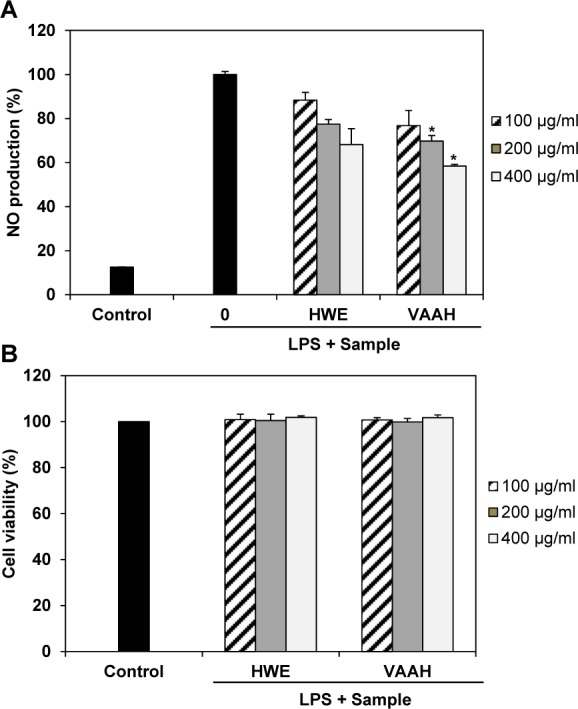
Effects of enzymatic hydrolysate of velvet antler (VAAH) on NO production (A) and cytotoxicity (B) in LPS-induced RAW 264.7 cells. Cells were pretreated for 1 h with different concentrations of hot water extract (HWE) and VAAH, LPS (1 μg/ml) was then added and cells were incubated for 24 h. Cytotoxicity was determined using the MTT assay. Each value is expressed as mean ± SE (*n*=3). Significant difference from the HWE group was identified at *p < 0.05 as analyzed via Duncan's multiple range test.

In this study, we evaluated potential anti-inflammatory effects of VAAH on NO production in LPS-stimulated RAW 264.7 cells. LPS markedly stimulated NO production in these cells compared to its effects in control cells. However, VAAH significantly inhibited NO production in LPS-stimulated cells in a dose-dependent manner. Furthermore, the inhibitory effect of VAAH on NO production was higher than that of hot water extracts (Figure 1A(Fig. 1)). In addition, VAAH did not have any cytotoxic effect on RAW 264.7 cells at all concentrations tested (Figure 1B(Fig. 1)).

Thus, the observed inhibitory effects are unlikely to be attributable to cytotoxic action. Our findings suggest that VAAH might play a role in inhibiting NO release. Previously, Suh et al. (2007[29]) showed that water extract of velvet antler possess anti-inflammatory activities in arthritic joints of mice that are associated with suppression of inflammatory mediators. The previous and present results indicate that velvet antler has anti-inflammatory activities. 

### Effects of VAAH on LPS-induced expression of iNOS and COX-2 proteins

**Figure 2:**
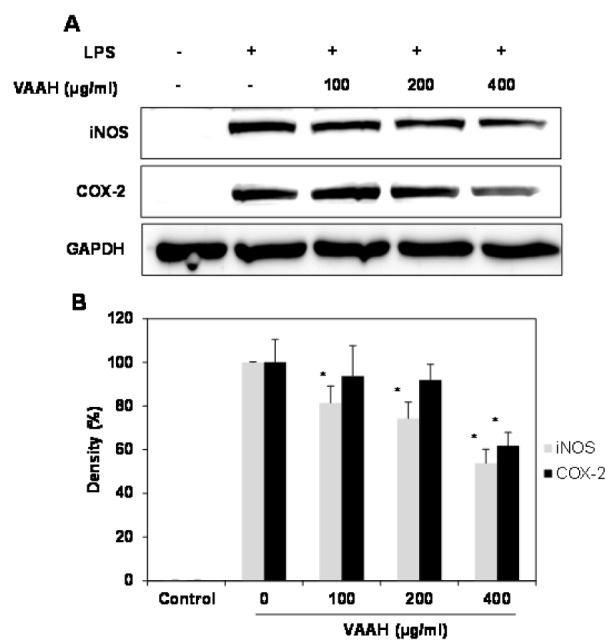
Effects of VAAH on LPS-induced iNOS and COX-2 protein expressions in LPS- RAW 264.7 cells. Cells were treated for 1 h with different concentrations of VAAH and then LPS (1 μg/ml) was added and incubated for 24 h. Equal amounts of cell lysates were subjected to electrophoresis and analyzed for iNOS and COX-2 expressions by Western blot. GAPDH was used as an internal control. (A) iNOS and COX-2 protein expression; (B) expression levels of iNOS and COX-2. Each value is expressed as mean ± SE (*n*=3). Significant difference from the only LPS-treated group was identified at *p < 0.05 as analyzed via Duncan's multiple range test.

Pro-inflammatory enzymes, including inducible nitric oxide synthase (iNOS) and cyclooxygenase-2 (COX-2), are capable of producing large amounts of NO, which, in turn, influences many chronic diseases associated with inflammation. We carried out Western blot analysis to determine whether the inhibitory effects of VAAH on the pro-inflammatory mediator NO were related to the modulation of iNOS and COX-2 expression. LPS-stimulated overexpression of iNOS and COX-2 proteins was reduced by VAAH in a dose-dependent manner compared to the expression seen in LPS-stimulated cells (Figure 2(Fig. 2)). These results suggest that the effect of VAAH on the inhibition of the release of NO as key mediators of inflammation may be attributed to expressional inhibition of iNOS and COX-2.

### Effects of VAAH on cell death and on intracellular ROS and NO generation in zebrafish 

**Figure 3:**
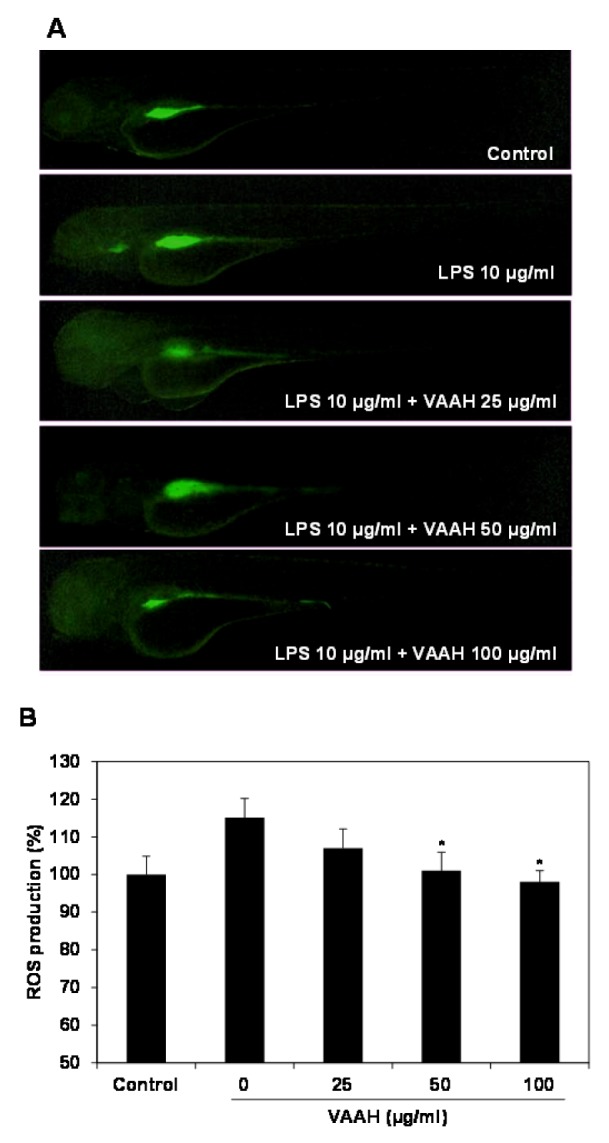
Inhibitory effect of VAAH on LPS-stimulated ROS production in zebrafish. The zebrafish embryos were pretreated with VAAH and exposed to LPS (10 µg/ml). Fluorescence micrographs of LPS-stimulated ROS generation in zebrafish embryos (A). The ROS generation level was measured by fluorescence intensity after staining with DCF-DA (B). Each value is expressed as mean ± SE (*n*=3). Significant difference from the only LPS-treated zebrafish was identified at *p < 0.05 as analyzed via Duncan's multiple range test.

**Figure 4:**
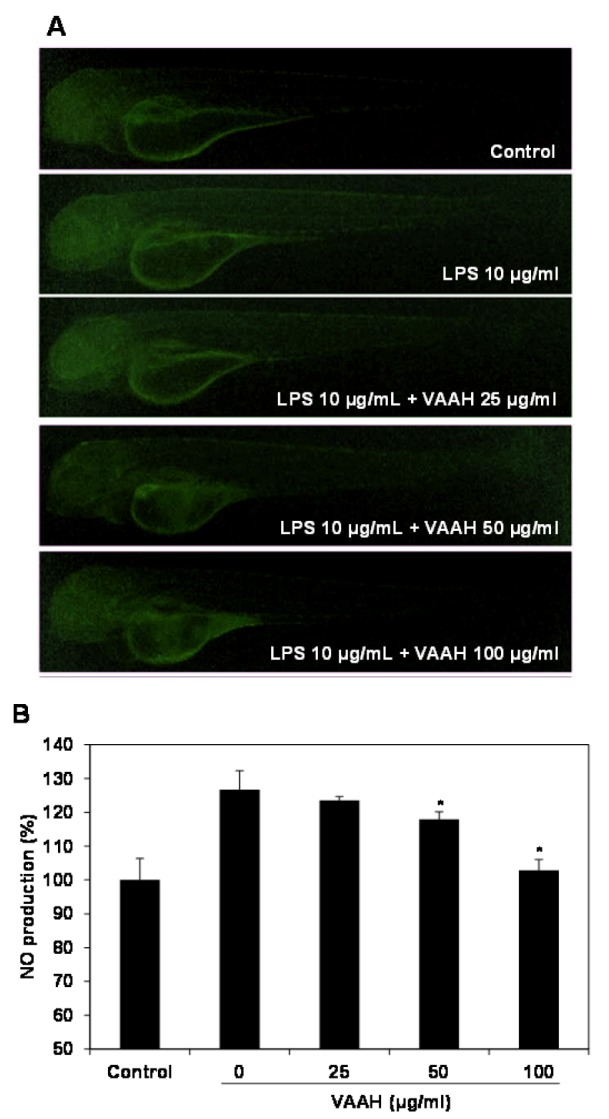
Inhibitory effect of VAAH on LPS-stimulated NO production in zebrafish. The zebrafish embryos were pretreated with VAAH and exposed to LPS (10 µg/ml). Fluorescence micrographs of LPS-stimulated NO generation in zebrafish embryos (A). The NO generation level was measured by fluorescence intensity after staining with DAF-FM-DA (B). Each value is expressed as mean ± SE (*n*=3). Significant difference from the only LPS-treated zebrafish was identified at *p < 0.05 as analyzed via Duncan's multiple range test.

Recent studies have reported that zebrafish was used to rapidly and simply assess the anti-inflammatory activity against LPS-stimulated inflammation *in vivo* (Park and Cho, 2011[23]). Therefore, in the present study, we investigated the anti-inflammatory effect of VAAH *in vivo* using zebrafish model. Intracellular ROS generation can be detected using oxidation sensitive fluorescent probe dye, 2',7'-dichlorodihydrofluorescein diacetate (DCF-DA) as the substrate. DCF-DA exhibits no fluorescence without ROS and becomes fluorescent upon interaction with ROS (Handa et al., 2006[7]). Zebrafish incubated with LPS showed that generation of ROS is significantly higher relative to the non-LPS treated zebrafish (negative control). Pretreatment with VAAH before LPS administration showed a significantly reduced ROS generation (Figure 3(Fig. 3)). This result suggests an inhibition of ROS generation by VAAH treatment. We also evaluated the inhibitory effect of VAAH on LPS-induced NO production in zebrafish by using the fluorescent probe dye diamino-fluorophore 4-amino-5-methylamino-2',7'-difluorofluorescein diacetate (DAF-FM DA). Transformation of DAF-FM DA by NO in the presence of dioxygen generates highly fluorescent triazole derivatives (Itoh et al., 2000[9]). Figure 4(Fig. 4) shows that NO level in zebrafish was significantly elevated by the LPS treatment as compared with the non-LPS treated zebrafish (negative control). However, the NO level in the VAAH-treated zebrafish was reduced significantly. The level of NO in zebrafish treated with LPS was 126.77 %, but pretreatment with 100 µg/ml of VAAH together with LPS exposure resulted in a lower NO level of 102.21 %. Acridine orange is a nucleic acid selective fluorescent cationic dye useful for apoptotic cells.

**Figure 5:**
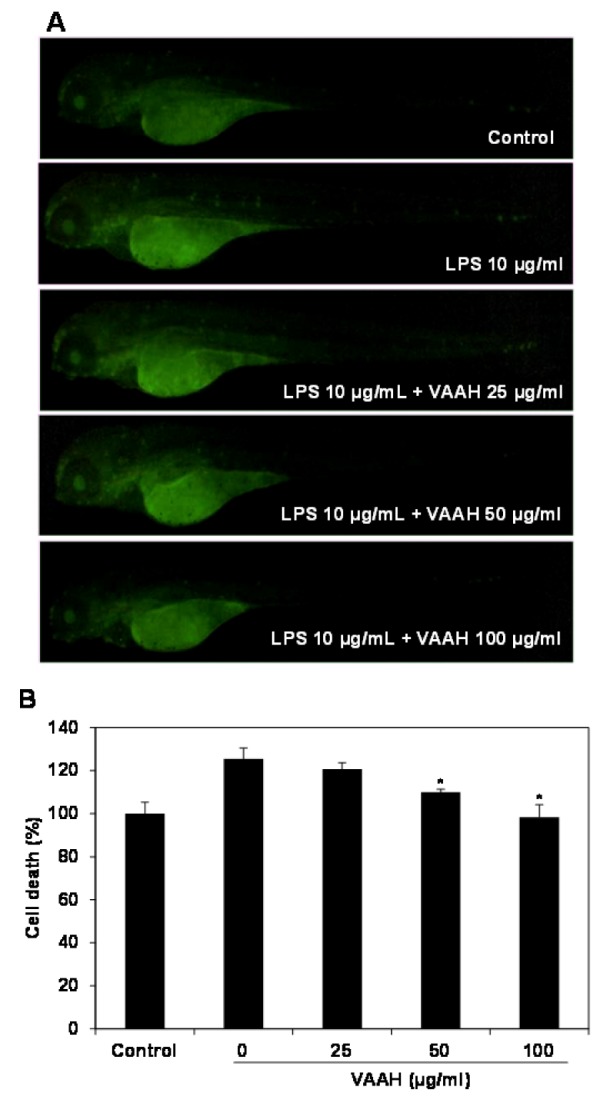
Protective effect of VAAH on LPS-stimulated cell death in zebrafish. The zebrafish embryos were pretreated with VAAH and exposed to LPS (10 µg/ml). Fluorescence micrographs of LPS-stimulated cell death in zebrafish embryos (A). The cell death level was measured by fluorescence intensity after staining with acridine orange (B). Each value is expressed as mean ± SE (*n*=3). Significant difference from the only LPS-treated zebrafish was identified at *p < 0.05 as analyzed via Duncan's multiple range test.

Lastly, Figure 5(Fig. 5) shows that cell death induced by LPS treatment was confirmed via acridine orange as 127 % fluorescence intensity. Cell death in zebrafish was significantly elevated by the LPS treatment as compared with non-LPS treated zebrafish (negative control). However, the LPS-induced cell death in VAAH-treated zebrafish was significantly reduced in a dose-dependent manner. Previous studies have indicated that increased ROS level induces oxidative stress which can result in the development of a variety of biochemical and physiological lesions. Such cellular damage frequently impairs the metabolic function and results in cell death and inflammation of tissues (Finkel and Holbrook, 2000[4]). 

In this study, LPS treatment was found to increase ROS generation in zebrafish and VAAH was found to effectively inhibit ROS generation. These findings indicate that VAAH alleviated inflammation and cell death by inhibiting ROS generation induced by LPS treatment. This outcome could explain potential anti-inflammatory activity of VAAH, which might have a beneficial effect during the treatment of inflammatory diseases.

In conclusion, the present study demonstrated that VAAH contained a considerable amount of bioactive components such as sialic acid, sulfated-GAGs, and uronic acid. Furthermore, VAAH exhibited a stronger anti-inflammatory action compared to the effects seen in the case of hot water extracts. VAAH also exhibited a potent anti-inflammatory effect in a zebrafish model. Therefore, enzymatic hydrolysis of velvet antler is an effective process to make antler-derived products more acceptable as health foods and nutraceuticals with increased bioactivity. In summary, velvet antler enzymatic hydrolysate can be used as a potential natural source of substances with a strong anti-inflammatory effect.

## Acknowledgements

This paper was supported by Konkuk University in 2014.

## Figures and Tables

**Table 1:**
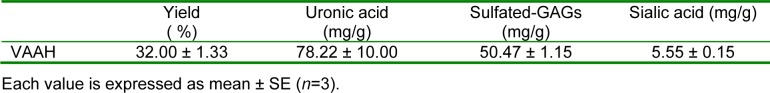
The extraction yield and bioactive component composition of velvet antler enzymatic hydrolysate (VAAH)
